# Study on Microstructure and Properties of Ni60A/WC Composite Coating by Alternating-Magnetic-Field-Assisted Laser Cladding

**DOI:** 10.3390/mi13050653

**Published:** 2022-04-20

**Authors:** Yuxu Zhu, Houming Zhou, Zixin Chen, Zeda Wang, Fangjia He, Caixing Xu

**Affiliations:** School of Mechanical Engineering, Xiangtan University, Xiangtan 411105, China; zhuyuxu@126.com (Y.Z.); zixincchen@163.com (Z.C.); wzd961324616@163.com (Z.W.); hfj980116@163.com (F.H.); 18373281071@163.com (C.X.)

**Keywords:** laser cladding, alternating magnetic field, microstructure, microhardness, wear property

## Abstract

Ni60A/WC composite coating is prepared on 45 steel substrate by alternating-magnetic-field-assisted laser cladding. We compare the effects of different magnetic field intensity on WC particle distribution, microstructure, phase composition, microhardness and wear; in addition, the mechanism of alternating magnetic fields on cladding layers is briefly analyzed. The results show that an alternating magnetic field can significantly homogenize the distribution of WC particles. WC particles at the bottom are stirred and dispersed to the middle and upper area of the laser pool. The distribution of WC in the bottom region 6 of the coating decreases from 19.1% to 10%, the distribution of WC in the bottom region 5 decreases from 46.46% to 33.3%, the WC distribution in the top region 1 of the coating increases from 0 to 7.7% and the WC distribution in the top region 2 of the coating increases from 8.08% to 12.2%. The stirring of alternating magnetic fields strengthens the solute convection in the laser pool, refines the snowflake-shaped carbide hard phase and improves the coating microhardness and wear property, and adhesive wear and abrasive wear decrease gradually with increasing magnetic field strength.

## 1. Introduction

As an advanced surface modification technology, laser cladding technology is equipped with excellent metallurgical bonding strength, a small heat-affected zone and a low dilution ratio. On the basis of this, laser cladding enjoys broad applications and is widely adopted in the aviation, shipbuilding, and petrochemical sectors. It has been widely considered by scholars at home and abroad as one of the current research hotspots [1,2,3].

The hardness and wear resistance of laser-cladded coatings can be prominently optimized by adding ceramic particles to the coating powder [4]. In industrial production, Ni-based alloys and WC ceramic particles are often mixed to endow a cladding layer with good performance. WC ceramic particles are equipped with high hardness, high temperature resistance and favorable wear resistance. Thus, in order to improve the hardness and wear resistance of a cladding layer, it is common to add WC ceramic particles to laser-cladded coating powder [5,6,7]. However, ceramic particles are, in general, concentrated at the bottom of the cladding layer, resulting in stress concentration, high cracking sensitivity, the uneven distribution of ceramic particles and poor performance improvement in the coating [8,9,10]. Therefore, the coating microstructure can be optimized through the even distribution of ceramic particles in the cladding layer, so as to further enhance its performance.

A. Ortiz et al. investigated the effect of improving WC distribution by increasing the content of WC particles in cladding powder. It was found that a coating with average WC concentration can be obtained by increasing the content of WC particles in the cladding powder. Even though WC particle distribution is made uniform through this method, it is not conducive to improving the coating quality and can incur cracking and porosity in the case of high WC particle content. Anandkumar et al. [11] investigated the distribution of SiC particles in the cladding layer of alusil alloy under different feeding rates from the perspective of process parameters. The results show that SiC particles are concentrated at the bottom of the laser pool under the feeding rate of 1 m/s, while they mainly stay at the surface of laser pool under the feeding rate of 5 m/s. Despite the varied distribution, SiC is still concentrated at the top of the coating rather than being evenly distributed.

An external field can affect the motion form of fluid, which can change energy transfer and material transfer during the laser cladding process. Thus, the addition of an external field is focused on by many researchers. Hu Yong et al. [12] studied the influence of applying an external steady-state magnetic field on the distribution of spherical WC particles. The results indicate that the external steady-state magnetic field can significantly inhibit the Marangoni convection in a laser pool. Most of the WC particles were concentrated on the upper surface and difficult to move to the inside of the laser pool. Even though the particle distribution was under control, WC particles were hindered from spreading inside the coating, which is adverse to optimizing coating performance. In terms of alternating magnetic fields, Fu et al. [13] investigated the effect of an alternating magnetic field on the mechanical properties and metallographic structure of a cladding layer. The results showed that convection in the laser pool was intensified by an alternating magnetic field, which broke the coarse dendrite arm, refined the grain, and made the surface structure increasingly dense and uniform.

To summarize, the experimental results are less than satisfactory through the adjustment of process parameters and the application of steady-state magnetic fields. An alternating magnetic field can intensify the convection, stirring the laser pool and ceramic particles inside and altering the distribution of ceramic particles in the pool. However, related research on using alternating magnetic fields to improve particle distribution are still limited. Therefore, we aimed to investigate the effects of alternating-magnetic-field-assisted laser cladding on the WC particle distribution, microstructure and properties.

## 2. Experimental Section

**Figure 1:**
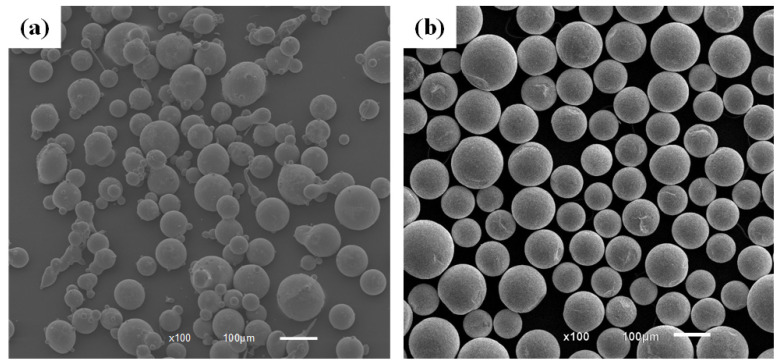
SEM images of (**a**) Ni60A powder and (**b**) WCPs.

**Figure 2:**
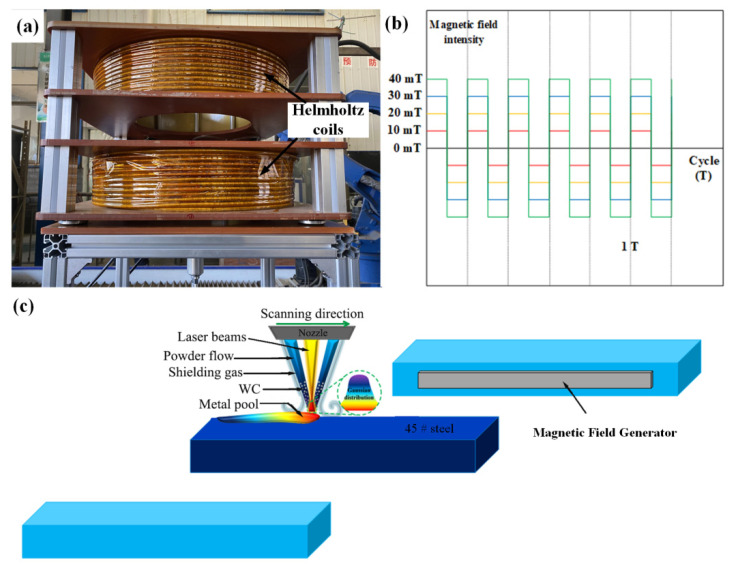
(**a**) Physical diagram of magnetic field generator; (**b**) schematic diagram of magnetic field waveform; (**c**) experimental schematic.

Here, 45 steel was utilized as the substrate in the experiment, and its major components are shown in Table 1. Ni60A powder, the main components of which are shown in Table 2, was mixed with spherical WC particles in a mass ratio of 4:1 and used as an experimental coating. The particle size of Ni60A powder and spherical WC particles was 100–270 mesh. The microstructure is shown in Figure 1. The laser used in the experiment was LDM3000-100. The processing parameters in the experiment were as follows: the laser power was 1400 W, the defocus was 0 mm, the scanning speed was 5 mm/s and the feeding rate was 15 g/min. As shown in Figure 2a, the magnetic field generator was composed of two Helmholtz coils in series. It could generate a vertical alternating magnetic field, in which the adjustable range of field frequency was 0–10 Hz, and that of field intensity was 0–45 mT. The parameters for the alternating magnetic field in the experiment were as follows: the frequency was 5 Hz, and the magnetic field intensities were 0 mT, 10 mT, 20 mT, 30 mT and 40 mT. Figure 2b shows the waveform of the magnetic field.

The JSM-6510LV scanning electron microscope was used to observe the coating structure. The Energy Dispersive Spectrometer (EDS) was utilized to analyze the element composition of the coating; the D/MAX-2500PC X-ray diffractometer was used to analyze the phase composition of the coating; the TMHV-1000 micro Vickers hardness tester was used to measure the microhardness of the cladding layer, with a load of 1.96 N and a duration of 15 S. The friction and wear properties of coating were investigated by using an HRS-2M reciprocating friction and wear tester under dry friction and with a 3.5% NaCl solution. The friction pair was a GCr-15 steel ball with 6 mm diameter, the friction load was 50 N, the grinding time was 30 min, the rotational speed was 600 r/min and the stroke was 5 mm.

## 3. Results and Discussion

### 3.1. Macrostructure

**Figure 3:**
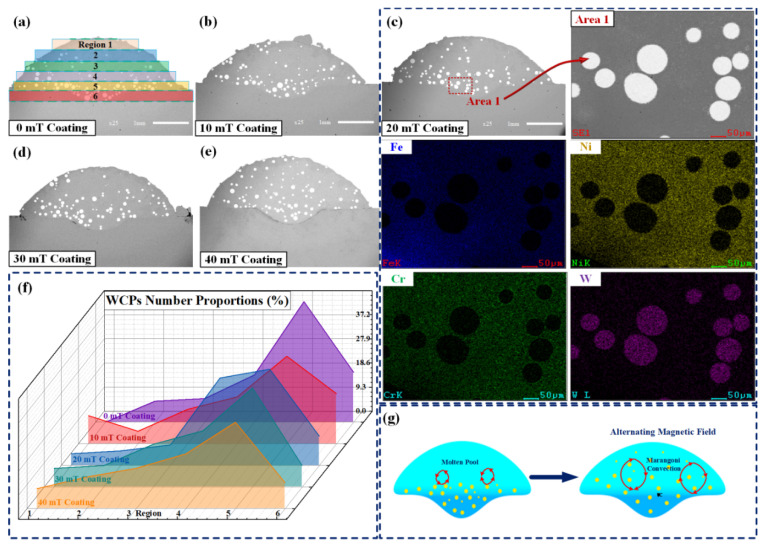
(**a**) The cross-sectional morphologies of coating of 0 mT; (**b**) the cross-sectional morphologies of coating of 10 mT; (**c**) the cross-sectional morphologies and the element map of Cr, Fe, Ni, W of coating of 20 mT; (**d**) the cross-sectional morphologies of coating of 30 mT; (**e**) the cross-sectional morphologies of coating of 40 mT; (**f**) the WCP proportions in particle volume ranges of coatings; (**g**) schematic diagram of the evolution mechanism of the WCP distribution.

Figure 3a–e presents the cross-sectional morphology of laser-cladded coating under different magnetic field intensities in SEM backscattering. The bottom of coating C with the magnetic field intensity of 20 mT was selected for EDS map scanning. The results can be found in Figure 3c, showing that white, bright, spherical substances are WC particles. Figure 3a shows that WC particles gathered at the coating bottom in the absence of magnetic field, while they were rarely distributed at the top and middle area. The distribution of WC particles varied greatly when an alternating magnetic field was applied, as shown in Figure 3b–e. In order to investigate the WC particle distribution in a detailed manner, the cross-section of the coating was evenly divided into zone 1, 2, 3, 4, 5 and 6 along the top to the bottom, as shown in Figure 3a. Additionally, we calculated the proportion of the number of WC particles in each zone to the number of those in the entire coating, as shown in Figure 3f. This shows that with the increase in magnetic field intensity, the proportion of WC particles in top zone 1 and 2 and middle zone 3 and 4 increases, while that of the bottom zone 5 and 6 decreases. As the magnetic field strength of the alternating magnetic field increases from 0 mT to 40 mT, the distribution of WC in the bottom region 6 of the coating decreases from 19.1% to 10%, the distribution of WC in the bottom region 5 decreases from 46.46% to 33.3%, the WC distribution in the top region 1 of the coating increases from 0 to 7.7%, and the WC distribution of the coating top region 2 increases from 8.08% to 12.2%. Particularly, the small WC particles move from the bottom of the coating to the middle and upper area. Small-sized WC particles are easily dispersed in the upper part of coating due to low gravity. With the increasing intensity of the alternating magnetic field, the WC particles originally concentrated at the bottom are distributed to the middle and top area, generating a uniform particle distribution in the coating. This is due to the alternating induced current generated in the laser pool under the action of the alternating magnetic field. The electromagnetic effect between the magnetic field and induced current generates an alternating electromagnetic force which intensifies the convection of liquid metal [14]. Given this, WC particles at the bottom are stirred and dispersed to the middle and upper area of the laser pool, which significantly optimizes their aggregation. Hence, WC particles are uniformly distributed in the coating, with its distribution variation shown in Figure 3g. In the absence of magnetic field, the convection will flow from high temperature to low temperature if only the Marangoni force is considered, showing a downward trend [15]. Given that in a short period of time the molten pool exists in liquid form, the solid WC particles are distributed at the bottom of the molten pool. When an alternating magnetic field is applied, a downward Lorentz force can be generated, which is in the same direction as the Marangoni force, thus intensifying the Marangoni convection [16]. The accelerated convection flow in the downward force stirs the WC particles at the bottom, resulting in particle dispersion to the middle and upper part of the molten pool. As is shown in Figure 3g, two rotational motions occur in opposite directions. The convection in the molten pool is strengthened owing to the application of an alternating magnetic field.

### 3.2. Microstructure

**Figure 4:**
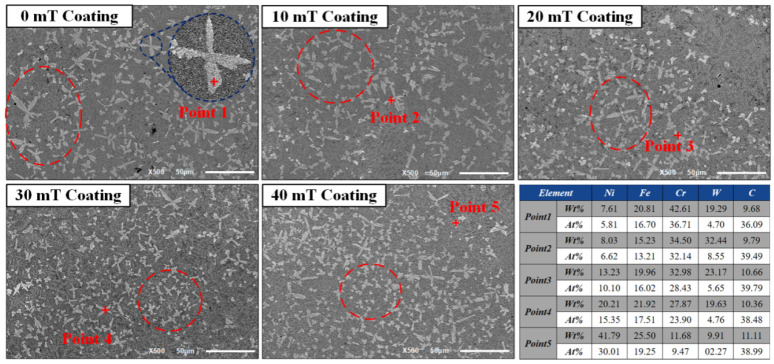
SEM images of middle regions of coatings and element composition at different points on the surface of coatings.

**Figure 5:**
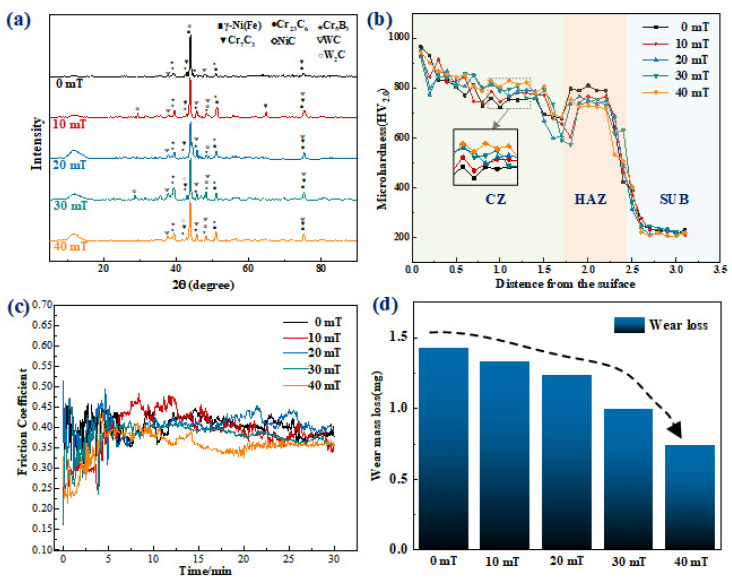
(**a**) XRD patterns of coatings; (**b**) microhardness of coatings; (**c**) friction coefficient curves of coatings; (**d**) wear mass loss of coatings.

Figure 4 shows the microstructure in the middle of laser-cladded coating under different magnetic field intensities, where we can see large, snowflake-shaped structures. EDS spot scanning was performed on the snowflake-shaped structure in Figure 4, as shown in Figure 4. EDS energy spectrum analysis showed that its main elements are Cr, Fe, Ni, W and C, which indicates composite carbide as the main component. Combined with XRD energy spectrum analysis, as shown in Figure 5a, the main phase compositions in the coating are mainly γ-Ni(Fe) solid solution and composite carbides and borides, such as Cr_23_C_6_, Cr_7_C_3_, Cr_5_B_3_, NiC, WC and W_2_C. Therefore, it was inferred that the snowflake-shaped structures are complex carbides formed by the eutectic reaction between dissolved WC particles and liquid metal [17,18,19]. Figure 4 indicates that the snowflake-shaped structure is large in the absence of alternating magnetic field, with large spacing and scattered distribution; with the increase in the applied magnetic field intensity, the snowflake-shaped structure gradually becomes smaller with the declining spacing and increasingly dense distribution. On the one hand, this is because the alternating magnetic field enhances the flow of solute in the laser pool, and WC particles are distributed more evenly in the coating shifting from the distribution at the bottom to the middle and upper area. Different from the heat at the bottom of laser pool, the heat in the entire laser pool is evenly absorbed by WC particles, which accelerates its dissolution. Additionally, the amount of snowflake carbide hard phase also increases [5]. On the other hand, as shown in Figure 3g, two rotating movements in opposite directions occur under the action of alternating magnetic field, enhancing the substance convection in the laser pool. This movement will “wash” the crystallized interface, break the primary dendrites and carbide hard phase, and refine the carbide hard phase structure [20].

### 3.3. Microhardness

Figure 5b shows the microhardness of cladding layers under different magnetic field intensities. The hardness of each coating is about 750 HV_0.2_ to 820 HV_0.2_ on average, which is higher than substrate hardness 210 HV_0.2_. The microhardness exhibits a salient downward trend because of the large temperature gradient at the top of coating during solidification and the dense structure formed by the rapid crystallization. Therefore, the hardness at the top of the coating is large, reaching 950 HV_0.2_ [21]. As observed from Figure 5b, in terms of the hardness in the middle of coating, the average coating hardness is the highest when the intensity of alternating magnetic field reaches 40 mT, while the average hardness is the lowest in the absence of the intensity of the alternating magnetic field. Generally, when the magnetic field intensity is higher, the corresponding coating hardness will be elevated as well, and as the intensity of alternating magnetic field reaches 40 mT, the coating will reveal relatively stable variation of hardness. However, there will be significant fluctuations in coating hardness in the absence of the alternating magnetic field. The higher the magnetic field intensity is, the more stable the variation that will occur in coating hardness. The hardness values over the middle of coating are stable, fluctuating less in this area. This is because, on the one hand, with the increasing intensity of the alternating magnetic field, the stirring in the laser pool becomes increasingly prominent. WC particles are more evenly distributed in the laser pool, absorbing more heat, promoting the formation of carbide hard phase in the cladding layer and strengthening the solid solution. On the other hand, WC decomposes when heated and reacts with the surrounding metal solution, promoting the growth of nuclei and accelerating the formation and nucleation of heterogeneous crystals. At the same time, unmelted WC particles can hinder the growth of dendrites and alter the formation of growth [22]. Therefore, with increasingly uniform distribution of WC particles, the grain can be refined to significantly improve the microhardness. The formation of a large number of high-hardness carbides can prominently improve the coating microhardness, which can effectively hinder the growth of dendrites, refine the grains, and strengthen the dispersion. The dispersion strengthening will obtain a better effect when the distribution of WC is more uniform in the coating. WC particles can realize the function of solution strengthening and dispersion strengthening. With growing magnetic field intensity, the WC content in the middle of the coating becomes higher, and the carbide hard phase precipitated in the middle of the coating increases. Furthermore, with the increase in magnetic field intensity, the carbide hard phase is “broken”, forming more and smaller carbide hard phases, and the average microhardness increases to realize dispersion strengthening [23]. Figure 5b shows that coating hardness decreases sharply in the area 1.3~1.7 mm away from the coating surface, because coarse dendrites are mainly concentrated at the bottom of the coating and feature low hardness. However, the area 1.8~2.2 mm away from the coating surface, which refers to the heat-affected zone, shows a rapid increase in hardness. This is because the heat is transmitted to the substrate during laser cladding, resulting in martensite phase transformation and quenching. It is observed that the coating hardness of the heat-affected zone is the highest in the absence of a magnetic field. The hardness of the heat-affected zone decreases with increasing magnetic field intensity. This is because in the absence of a magnetic field, a large number of WC particles precipitate to the bottom area, and many carbon elements penetrate into the substrate, resulting in the high hardness of the heat-affected zone. With the increase in magnetic field intensity, the distribution of WC particles in the middle area increases, the WC particles at the bottom decrease, the carbon elements penetrating into the substrate decrease and the hardness of the heat-affected zone decreases.

### 3.4. Wear Properties

Figure 5c,d show the friction coefficient curve and wear loss of the coating under dry friction with different magnetic field intensities under 50 N load. The friction coefficient curve is divided into two periods: the initial wear period and the stabilization period. From 0 to 4 min, the friction pair starts being in contact with the coating surface, characterized by instability, rough friction surface and a violent wear process. Thus, the friction coefficient exhibits fluctuating changes [24,25,26]. After more than 4 min, the wear on the cladding layer becomes stable, and grooves on the friction surface are gradually flattened. With the expansion of the contact surface and increase in interface temperature, the cladding layer is slightly worn during this period without much abrasive dust such as particles and with a small amplitude of friction coefficient change. Figure 5c shows that as the intensity of the alternating magnetic field increases, the friction coefficient tends to decrease gradually with smaller fluctuation. In the absence of an alternating magnetic field, the average friction coefficient is 0.43, with significant variation in the friction coefficient. The friction coefficient is 0.35 and stable in general when the intensity reaches 40 mT, with less variation in the friction coefficient. Figure 5d shows that as the intensity of the alternating magnetic field increases, the wear loss declines. Additionally, the wear weight loss is only 7.35 × 10^−4^ g in the case of 40 mT magnetic field intensity.

**Figure 6:**
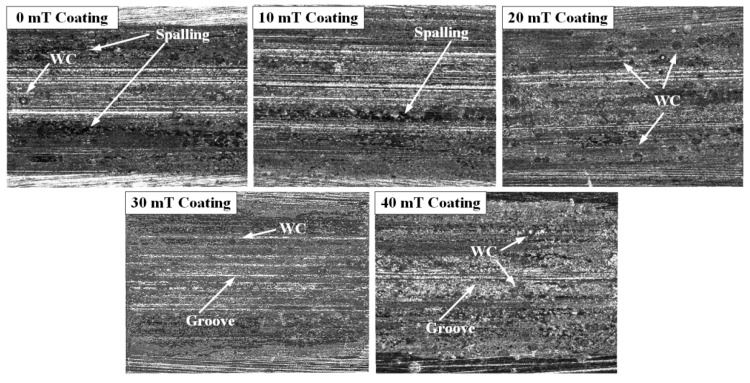
The wear morphology of the coatings.

Figure 6 shows the wear morphology of the coating under different magnetic field intensities. In the absence of an alternating magnetic field, the coating surface exhibits severe wear with shallow spalling and deep grooves. This is because most of the WC particles gather at the bottom of coating in the absence of an alternating magnetic field. Due to the uneven distribution of WC particles, the particles may be densely located in a certain area, resulting in ill combination with the coating. Consequently, WC particles may easily spall during the friction process, leading to abrasive wear and grooves on the friction surface. As can be observed from Figure 6, when the magnetic field intensity increases, there are no obvious furrows and large spalling layers, but only some small spalling pits, indicating the decreasing adhesive and abrasive wear [27,28]. This is because with the growing intensity of the alternating magnetic field, the WC particles are uniformly distributed. Therefore, the coating microstructure becomes finer and produces a stronger imbedding force. Additionally, WC particles are less prone to spalling, which reduces the wear. On the other hand, the stirring of the alternating magnetic field on WC particles facilitates the heat absorption in the laser pool, accelerates the decomposition of WC particles, promotes the formation of carbide hard phase in the melt layer, improves the coating hardness and optimizes the wear resistance of the coating simultaneously.

## 4. Conclusions

1. In terms of stirring in the laser pool, the alternating magnetic field intensifies the convection of liquid metal, stirring WC particles at the bottom and dispersing them to the middle and upper part, leading to a uniform distribution of WC particles.
2. The alternating magnetic field significantly refines the carbide hard phase structure, and the phase composition of the coating does not change.
3. The alternating magnetic field promotes the melting of WC particles, enhances the solution strengthening and dispersion strengthening and improves the coating hardness.
4. The wear resistance of the coating is optimized when the alternating magnetic field is applied. As major wear forms, the adhesive and abrasive wear will gradually decrease as the magnetic field intensity increases.

## Figures and Tables

**Table 1:** Chemical compositions of the 45 steel substrate (wt%).

Composition	C	Cr	Mn	Si	Ni	Fe
**Percent**	0.42~0.50	≤0.25	0.50~0.80	0.17~0.37	≤0.25	The rest

**Table 2:** Chemical compositions of the Ni60A substrate (wt%).

Composition	C	Cr	Si	B	Fe	Ni
**Percent**	0.60~1.00	14.00~16.00	3.00~4.50	2.50~3.50	≤0.25	The rest

## Data Availability

Not Applicable.
